# Assessment of a Novel Instrument Measuring Perceived Physical Education Teachers’ In-Class Skills

**DOI:** 10.3390/bs13010042

**Published:** 2023-01-04

**Authors:** Armando Cocca, Nellie Veulliet, Clemens Drenowatz, Katharina Wirnitzer, Klaus Greier, Gerhard Ruedl

**Affiliations:** 1 Department of Sport Science, University of Innsbruck, Fürstenweg 185, 6020 Innsbruck, Austria; 2 Division of Sport, Physical Activity and Health, University of Education Upper Austria, Kaplanhofstraße 40, 4020 Linz, Austria; 3 Department of Research and Development in Teacher Education, University College of Teacher Education Tyrol, Pastorstraße 7, 6010 Innsbruck, Austria; 4 Research Center Medical Humanities, Leopold-Franzens University of Innsbruck, Innrain 52, 6020 Innsbruck, Austria; 5 Division of Physical Education, Private Educational College (KPH-ES), Stiftshos 1, 6422 Stams, Austria

**Keywords:** professional characteristics, personal characteristics, teaching-learning process, adults, structural validity, active behaviors

## Abstract

Physical Education (PE) teachers’ professional and personal skills may not only affect the quality of the teaching-learning processes in PE, but also individuals’ future active/inactive behaviors. The aim of this study is to examine the structure of a pool of items developed for measuring individuals’ perception of such skills in PE teachers. Exploratory Factorial Analysis and a following structural modeling test on data collected from 660 participants suggest a two-factor structural model for the 10 items considered (χ^2^ = 191.155; df = 34; CFI = 0.953, and SRMR = 0.0529), with good internal consistency for both factors (factor 1: alpha = 0.879; omega = 0.878; factor 2: alpha = 0.850, and omega = 0.858) and the overall instrument (alpha = 0.892; omega = 0.895). The final “Teachers’ Personal and Professional Skills Questionnaire” is a valid instrument that may be used alone or in combination with other instruments for the analysis of the quality of teaching-learning processes in PE environments and its impact on individuals’ behaviors regarding physical activity in their adult life.

## 1. Introduction

Physical education (PE) is an important contributor to the development of motor skills, social inclusion, as well as for the promotion of health in young people and their families [1,2]. The role that PE plays in increasing youth’s total level of physical activity (PA) is fundamental, and the association between physical exercise carried out in the school context, the level of daily healthy PA [3], the promotion of a healthy physical and mental growth [4,5,6], and the prevention of potential health threats is well known [7,8]. Furthermore, PE could provide a substantial portion of PA that allows students to reach the minimum weekly PA recommended to have benefits on their health [9]. This is relevant considering that most young people do not meet the minimum requirements for healthy physical exercise [10]. However, benefits are obtained from PE only if its quality is high [11], while lower-quality PE may lead to a greater risk of developing harmful habits, such as high sedentary lifestyles [12].

One of the cornerstone elements for quality PE is represented by the PE teachers [8]. In particular, teachers may influence students’ in-class experience in two different ways: based on their professional preparation [13], and through their interpersonal skills [14]. Regarding teachers’ professional characteristics, Vergara-Torres et al. [15] mention that their task presentation and quality of feedback may influence students’ perceived autonomy and motivation to be active; teachers’ personal commitment and dedication are also essential aspects to be considered when evaluating the success of an educational program [16], along with teachers’ self-confidence and perceived efficacy [17]; the importance of other professional aspects, such as teachers’ knowledge and preparation, has been highlighted in previous studies on general education and PE, as well [18,19]. In terms of social/personal skills, it has been demonstrated that a positive teacher-student interaction, as well as PE teachers’ proper behaviors, may enhance students’ attitudes during the lessons [20]; teachers showing positive behaviors in the classroom also help students fulfill their social and emotional needs [21], in addition to having an impact on their enthusiasm and academic achievements [22]. Furthermore, building a positive teacher-student relationship triggers higher engagement in the proposed lesson activities [23]. Mameli et al. [24] add that interpersonal behaviors, such as a fair attitude towards all students, help create a better climate and foster students’ willingness to express themselves. Contrary to that, when teachers behave in a more negative way, students tend to become less interested and active in the classroom [25]. For instance, impatient teachers are seen by students as less willing to take time to explain things and less prone to listen to them [25]. In addition, low teachers’ emotional skills may not only affect their students’ emotions directly, but they may also have a comparable impact as low pedagogical skills [26].

Teachers’ ability to both deliver proper teaching and connecting with the students at a social level does not only affect their pupils in the short term but also has an impact on their future lives as adults. In a study by Doll et al. [27], poor teaching quality, along with teachers’ inability to emotionally help their students, are among the main reasons for individuals to drop out of their studies later in their careers. Jackson [28] adds that the impact of teachers on their students is more significant on long-term behaviors than it may be immediately, for instance, on their grades. This also seems to be confirmed with regard to students’ future financial success, as it has been suggested that improved teaching may predict increased earnings later in life [29]. A report on teachers’ short- and long-term effectiveness adds that quality teaching at early educational levels may positively affect not only future academic success, but also quality of neighborhood of residence and even parenthood [30]. All of these long-term outcomes may be due to students’ increased self-confidence thanks to their teachers’ skills both in terms of teaching and supporting their pupils at a social-psychological level [31]. This is also true in the field of PE, where the impact of teachers on their students’ active lifestyle and risk of chronic diseases has been stressed in previous studies. For instance, the influence of the quality of the teaching in PE was already associated with adult lifestyle at the beginning of this century [32]. PE teachers’ quality characteristics are highlighted as a cornerstone for higher active behaviors and lower health risks both immediately and in the long term [33]. This is further confirmed by Wintle [34], who discusses PE teachers’ influence on the learning process and its consequences in adulthood. Ladwig et al. [35] report that PE teachers’ qualities and social relations with their students have an impact on their memories of PE in adulthood, this having a significant association with their attitude towards PA, intention to be active, and even on sedentary behaviors.

In spite of the above-mentioned evidence on the key role played by teachers’ internal and interpersonal characteristics, there is a lack of specific instruments collecting data on them in the area of PE. Previous literature shows different strategies, such as (a) a focus on only specific characteristics; for instance, Vergara-Torres et al. [15] asked individuals to report their perception of teachers’ ability to present tasks or deliver feedback. Other authors focused on the planning/delivering skills of PE teachers, and used ad hoc instruments for the particular purpose [36], or on students’ perception of their PE teachers’ ability to support autonomy [37]; (b) the analysis of teachers’ self-perception of their in-class abilities; Cocca and Cocca [38], for instance, delivered an updated version of a self-efficacy questionnaire focused on teachers’ abilities to respond to in-class occurrences in PE, while others have worked with self-efficacy in different PE conditions [39]; and (c) the assessment of students’ expectations from PE teachers, rather than the perception of their current or past ones [40]. It is clear that there is a gap in this area, and that instruments covering both types of skill sets (professional and interpersonal) required from a PE teacher could provide important help in the study of short- and long-term active behavior adherence, as well as in the assessment of quality PE. Within this field of study, Brettschneider et al. [41] created a pool of twelve questions for measuring such teachers’ skills within the framework of a larger project on sport in schools in German-speaking countries. The items in the questionnaire were generated after an extensive analysis of PE, which included observations and interviews with students, teachers, parents, and experts in the field, as well as being based on the previous literature of PE teachers. The questions consisted of different adjectives describing teachers professional and personal qualities, such as “just”, “sympathetic”, “professional”, or “committed”. Students were asked to rate each teacher’s characteristics using a semantical differential method, with grammatically opposite adjectives at each end of the scale (for instance, from “unfair”—extreme left, to “just”—extreme right). Although this pool of questions has already been used with the purpose of assessing students’ perception of their teachers [41], a comprehensive analysis of its internal and structural validity has never been carried out. Considering the potential implications of the use of such tools both in terms of evaluation of an educational system and the development of future teachers, and the long-term impact on individuals’ lives, as well as the above-underlined lack of instruments for these variables, it seems essential to examine their scientific robustness with the aim of introducing a potentially powerful tool for both researchers and practitioners in PE. Therefore, the aim of this study is to assess the psychometric parameters of the pool of questions on teachers’ skills developed by Brettschneider et al. [41], with particular attention given to its internal structure and reliability.

## 2. Methodology and Methods

### 2.1. Design

The design of this study is quantitative, non-experimental, and observational.

### 2.2. Sample

A total of 660 active adults (401 women, 259 men; mean age = 38.80 ± 17.42) with an average height of 172.42 ± 8.85 cm and an average weight of 68.17 ± 13.14 kg (BMI = 22.81 ± 3.20) were recruited using a convenience sampling approach from a population of individuals attending courses offered by the University Sports Association of the researchers’ institute. The decision to selecting adults was made with the aim of using the final questionnaire as a recall instrument to be associated with adults’ exercise-related outcomes, such as PA and sedentary behaviors, or motivation to be active. Participants’ educational backgrounds consisted of completed bachelor studies (57.6%), high school diploma (32.9%), and other high school/secondary studies (8.4%).

Formal approval from the Ethical Committee of the researchers’ institution had been previously provided for all questionnaire-based research within the faculty (certificate of good standing n. 73/2021). Additionally, informed consent was given by participants prior to filling out the questionnaire.

### 2.3. Instruments

The pool of questions created by Brettschneider et al. [41] originally included 12 pairs of adjectives, each of them describing a different quality of teachers. Despite the original instrument being built with a semantic differential approach, the research team decided to rearrange the response method into a Likert-type scale, from 1 (the adjective does not describe the teacher at all) to 5 (the adjective fully describes the teacher), and selecting only one adjective for each given pair (for instance, “just” for the pair “just-unfair”). This change is due to the fact that Likert scales are the most used to collect data in research in educational sciences [42]; therefore, contrasting scores from this instrument with those from other tools in the educational field may become easier in future studies. Also, Artino et al. [42] suggest the use of 5- or 7-point Likert scales; this, together with the fact that the academic grading in the education system where this research was carried out ranges from 1 to 5, may make the choice of a 5-point scale easier to understand by the respondents. An overview of the final pool of questions (adjectives) is provided in Table 1 below.

The data collection was carried out during several consecutive days, and questionnaires were filled electronically by means of online survey software. The online layout included a brief introduction to the study, and respondents were instructed to recall their PE classes during school and (in case of having had more than one PE teacher during their educational path) to refer to the PE teacher they had for the longest time.

## 3. Data Analysis

The first step consisted of descriptive analysis and the recodification of the scores from negative adjectives. The questionnaire’s parameters were then tested using both IBM SPSS 26 and IBM Amos 22 software. Firstly, an Exploratory Factorial Analysis (EFA) with a Principal Components extraction method and Varimax rotation with Kaiser normalization was carried out on a smaller randomly selected sub-sample of 200 participants [38], so as to assess whether the adjectives could be grouped together. The initial data examination also included the assessment of correlations between adjectives by means of Spearman Correlation analyses in order to check for multicollinearity issues. Internal consistency analyses were carried out by means of Cronbach’s alpha and McDonald’s omega, which were applied to each factor separately. For both alpha and omega, values above 0.70 are considered acceptable, whereas they are good if above 0.80 [43,44]. Based on the outcomes of the EFA, the structural validity of the model with the remaining portion of the sample (n = 460) was verified by means of Confirmatory Factorial Analysis (CFA) using the Maximum Likelihood estimation method [45]. The model was scrutinized with the following goodness of fit indexes: Comparative Fit Index (CFI) and Standardized Root Mean Square Residual (SRMR). Cut-off points for these indexes were set at 0.95 or higher, and 0.09 or lower, respectively, following the guidelines provided by Hu and Bentler [46]. Additionally, based on the recommendations of Fabrigar [47] and Collier [48], modifications of the initial model were based on the evaluation of item loadings (not lower than 0.50) and Standardized Residual Covariances (SRC, not higher than 2).

## 4. Results

The EFA detected two main factors for the pool of adjectives, which explained 63.9% of the total variance. The first factor included adjectives describing teachers’ interpersonal qualities, for instance, “sympathetic”, “friendly”, or “just”; loadings for the adjectives in this factor ranged between 0.612 and 0.813. The second factor comprised adjectives related to professional skills, such as “competent”, “prepared”, or “athletic”, with loadings ranging from 0.678 to 0.815. A summary of the results from EFA is shown in Table 2 below.

Spearman correlations between the items included in the first factor were all statistically significant (*p* < 0.001). Correlation coefficients remained in the interval from 0.374 to 0.634; however, they were higher for the adjectives “sympathetic” and “friendly” (r = 0.748). Correlations between the adjectives in the second factor were also highly significant (*p* < 0.001), with values of correlation coefficients between 0.331 and 0.604.

Internal consistency analyses showed good scores for both factors (factor 1, alpha = 0.912, and omega = 0.912; factor 2, alpha = 0.850, and omega = 0.858).

**Figure 1:**
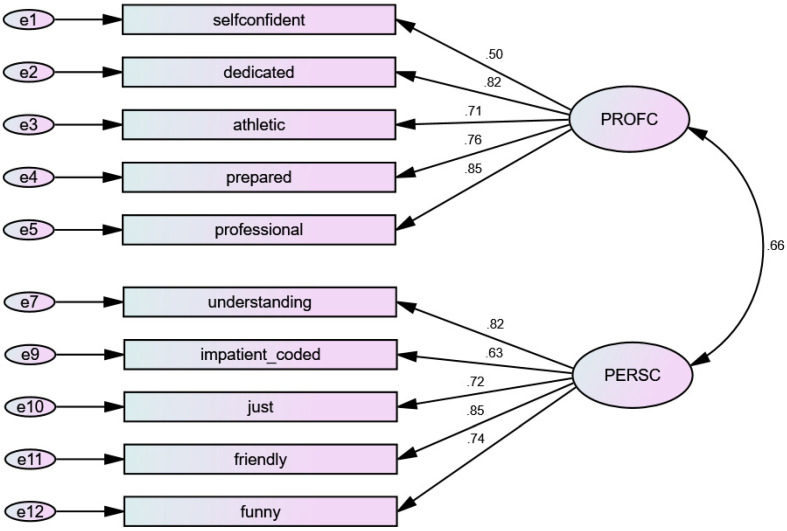
Final structural model for the pool of adjectives describing physical education teachers’ characteristics. Note. PROFC = Professional Characteristics; PERSC = Personal Characteristics.

A structural validity analysis based on the model suggested by EFA showed insufficient scores in the fit indexes due to a too low CFI value (χ^2^ = 508.828; df = 53; CFI = 0.903, and SRMR = 0.0628). Considering the outcomes from the previous correlation analysis, the model was tested after removal of (alternatively) sympathetic/friendly, whose correlation was too high. Removing “friendly” worsened the model fit; however, CFA after elimination of “sympathetic” delivered a slightly improved model, yet a still insufficient CFI score (χ^2^ = 402.399; df = 43; CFI = 0.906, and SRMR = 0.0628). After the revision of the SRCs and the subsequent removal of the adjective “moody” (SRC > 5.0), the model showed good fit indexes (χ^2^ = 191.155; df = 34; CFI = 0.953, and SRMR = 0.0529). The final model (Figure 1) was therefore composed of factor 1 (understanding, impatient, just, friendly, and funny), with good internal consistency after removal of the above-mentioned adjectives (alpha = 0.879; omega = 0.878), and factor 2 (self-confident, dedicated, athletic, prepared, and professional).

Finally, the analysis of the internal consistency for the whole pool of adjectives showed good scores for both alpha (0.892) and omega (0.895).

## 5. Discussion

The aim of this study was to evaluate the validity of a recall questionnaire on individuals’ perception of PE teachers’ personal and professional characteristics that was previously created in the framework of a larger scientific project on exercise in the school context.

In line with the suggested structure reported for such a project [41], in our study the questionnaire presents two correlated factors, one referring to teachers’ personal skills, such as their patience, their fairness, or their empathy with students in the classroom, and the second comprising characteristics of their professionalism, such as preparation, pedagogical skills, or their commitment to teaching. This internal division reflects the theoretical stream supporting the impact of teachers’ professional [13] and internal skills [14] on the successful development of the teaching-learning processes.

One of the peculiarities of the pool of adjectives analyzed in our study is the use of the adjective “athletic”, which, despite perhaps not commonly being seen as a required skill with a potential impact on students’ in-class experience, is shown to statistically fit in the overall model within the set of professional skills. Particularly in PE, i.e., the focus of the present study, being fit and athletic in order to represent a proper role model for students indeed seems to have an influence on students’ activity and motivation during PE lessons [49]. The importance of athleticism in individuals’ perception of their PE teachers is also pointed out by Sözen and Korur [50] in a study on middle school students, whose most frequent description of them was “athletic, physical force”, even more common than being referred to as “a good person” or “a guide”. According to González-Calvo et al. [51], being athletic is not only an essential element for the professionalism of PE teachers, but is also necessary for effectively transmitting health messages to their students. Thus, the inclusion of such an aspect in a tool assessing PE teachers’ characteristics may be understandable and justified.

The initial analysis on the pool of adjectives highlighted the risk of multicollinearity for the terms “sympathetic”/“friendly”. This may be evident by analyzing the meaning of the terms according to official dictionaries: the Collins English Dictionary defines “sympathetic” as someone supportive, caring, and more generally friendly to someone [52]; in a similar manner, the corresponding German words are also associated in the Digitales Wörterbuch der deutschen Sprache (Digital Dictionary of the German Language) [53]. Therefore, it is possible that the respondents would not differentiate enough between these adjectives and rated both terms in the same way. Multicollinearity may lead to problems when testing the robustness of a structural model, reducing the reliability of the related regression coefficients [54]. Grewal et al. [55] suggest that, although multicollinearity does not always lead to issues in the interpretation and validity of the outcomes in structural equation modelling, assessing in which way the estimates of the tested model change after the exclusion of the variable/s provoking the issue may be considered a good practice. If the model improves after removal of said variable/s, it is recommended to confirm the exclusion of one or more of the involved items [55]. In our case, after sequentially removing the adjectives “sympathetic” and “friendly”, the indices of goodness of fit improved for the model, including the latter and eliminating the former. The removal of “sympathetic” also contributes to reducing the final length of the questionnaires, which is known to help increase the response rate [56] at the same time as it positively affects respondents’ fatigue [57].

Based on our findings and the literature describing teachers’ sets of skills impacting the educational processes, the tested instrument may be considered scientifically robust, with a two-factor structure composed of a “teachers’ professional skills” dimension (five items) and a “teachers’ personal skills” dimension (five items). The two factors are correlated and together compose the “Teachers’ Personal and Professional Skills Questionnaire” (TPPS-Q), describing the perception individuals have of their PE teachers’ set of internal and external assets required for them to have an impact on the educational experience in a given PE classroom setting.

## 6. Limitations

The main limitation of this study lies in the fact that it requires participants to recall their PE teachers’ characteristics from previous years. Although asked to specifically focus on the teacher they had for the longest time during their school period, their perceptions might have been affected in a similar manner as in other recall-based studies, for instance due to memory recall errors [58]. Nonetheless, such errors tend to assume importance if the outcomes of the data analyses are used to draw conclusions on variable associations, such as cause-effect relationships [59], which, instead, are not considered for the purpose of our work. Another limitation is the fact that this study only focuses on structural and internal validation of the presented instrument, not carrying out other useful analyses, such as construct validity, predictive criterion-related validity, or test-retest reliability. In the future, it is recommended that the parametric analysis of the TPPS-Q be expanded to other populations (for instance, students from different educational levels, who would report their current experience in PE rather than recalling it from a past time), to examine potential sex differences, to carry out other instrument validation processes, and to include potential influencing factors as covariates, such as teachers’ years of professional experience.

## 7. Conclusions

The analysis of the structure and internal consistency of the TPPS-Q confirms that this instrument could be used in research focused on the evaluation of PE teachers’ characteristics that may improve students’ learning and classroom climate in PE and potentially affect their active behaviors during adulthood. As a consequence, future research may propose, on the one hand, the completion of the validation process by means of further analyses; and on the other hand, the use of the TPPS-Q in synergy with other scientific instruments, such as accelerometry for the study of the amount and intensity of weekly PA, or questionnaires on motivation towards PE/PA. This may contribute to establishing the potential mediating role of such teachers’ skills on the structuring of active habits in adults, which are essential in enhancing individuals’ lifestyles and health in the short and long term. Furthermore, it may also help educational systems and local/national administrations to better tailor the programs in physical education teacher education (PETE) at the tertiary education level so as to prepare future teachers not only in terms of professional abilities, such as pedagogical strategies and task presentation, but also in terms of their attitude towards students and content during the carrying out of PE lessons. However, we need to emphasize that, based on the typical limitations that come with recall instruments, the validation of this instrument in student populations is also recommended. This could successively allow for the implementation of the tool in longitudinal studies in which current teachers’ skills and PA habits are tracked over the years from youth to adulthood, leading to an even better overview of the connection between school PE and adult active lifestyle. Finally, the study of complex explanatory models including former PE experience and current PA habits should also consider the potential influence of factors influencing adults’ choices, such as occupation type (for instance, jobs that imply physical effort compared to office jobs), or family status (for instance, the number of children, etc.).

## Figures and Tables

**Table 1:** Pool of adjectives describing physical education teachers’ characteristics included in the tested structural model.

English Adjectives	German Adjectives
Athletic	Sportlich
Dedicated	Engagiert
Friendly	Freundlich
Funny	Humorvoll
Impatient	Ungeduldig
Just	Gerecht
Moody	Launisch
Prepared	Vorbereitet
Professional	Fachlich gut
Self-confident	Selbstsicher
Sympathetic	Sympathisch
Understanding	Verständnisvoll

**Table 2:** Outcomes of the Exploratory Factorial Analysis on the pool of adjectives describing physical education teachers’ characteristics (Principal Components extraction method; Varimax rotation with Kaiser normalization).

Adjective	Factor 1	Factor 2
Impatient	0.813	
Understanding	0.802	
Friendly	0.794	
Sympathetic	0.756	
Moody	0.753	
Just	0.693	
Funny	0.612	
Athletic		0.815
Professional		0.777
Prepared		0.741
Dedicated		0.738
Self-confident		0.678

## Data Availability

The data presented in this study are available on request from the corresponding author [AC]. The data are not publicly available due to privacy restrictions.
